# Healthcare Provider Testing Practices for Tinea and Familiarity with Antifungal-Drug-Resistant Tinea—United States, 2022

**DOI:** 10.3390/jof8080831

**Published:** 2022-08-09

**Authors:** Kaitlin Benedict, Karen Wu, Jeremy A. W. Gold

**Affiliations:** 1 Division of Foodborne, Waterborne, and Environmental Diseases, National Center for Emerging and Zoonotic Infectious Diseases, Centers for Disease Control and Prevention, Atlanta, GA 30329, USA; 2 Epidemic Intelligence Service, Centers for Disease Control and Prevention, Atlanta, GA 30329, USA

**Keywords:** tinea, ringworm, dermatophytosis, diagnosis, drug resistance, fungal, primary health care, United States

## Abstract

Tinea, a dermatophyte fungal infection, is a common outpatient complaint that is easily misdiagnosed by visual inspection. Antifungal-drug-resistant tinea is an emerging global public health problem, with several cases reported in the United States. We analyzed data from a Spring 2022 web-based survey of healthcare provider attitudes and practices. Among 1500 healthcare providers, only 20.1% reported typically using diagnostic testing for tinea, and 19.5% reported clinical experience with drug-resistant tinea. Drug-resistant tinea may be more widespread than previously recognized. However, the low frequency of diagnostic testing indicates potential misunderstanding or misdiagnosis of drug-resistant tinea and missed opportunities to detect drug-resistant cases.

## 1. Introduction

Tinea, also called ringworm or dermatophytosis, is a common infection of the skin, hair, or nails caused by dermatophyte fungi [1,2]. The infection can spread by fomites, between people, and between people and animals. The classic tinea lesion is an erythematous, raised, scaly annular rash with central clearing. The sites typically affected include feet (tinea pedis), hands (tinea manuum), groin (tinea crutis), scalp (tinea capitis), and other body sites (tinea corporis). Tinea can be easily misdiagnosed by visual inspection alone [3,4], potentially leading to inappropriate treatment, worsening of symptoms, or secondary bacterial infections. Available methods to confirm a diagnosis of tinea include direct microscopy, fungal culture, and fungal polymerase chain reaction. Routine diagnostic testing is generally only recommended for suspected tinea of the scalp and nails [1]. Healthcare provider (HCP) testing practices for tinea have not been well-described but may be increasingly important to understand given the global emergence of antifungal-drug-resistant tinea, a public health threat [5]. Although the extent of this problem in the United States is unclear, several US cases of antifungal-drug-resistant tinea have been reported [6,7]. To help inform tinea diagnosis and prevention efforts, we assessed HCP self-reported testing practices for tinea and familiarity with drug-resistant tinea.

## 2. Materials and Methods

We analyzed data from the Spring 2022 DocStyles survey, a web-based survey of HCP attitudes and practices commissioned by Porter Novelli Public Services and conducted by SERMO during 16 March to 4 May 2022. We analyzed two questions: “What methods do you typically use to diagnose patients with tinea (ringworm) on initial presentation?” and “Before this survey, were you familiar with reports of drug-resistant tinea (ringworm)?” Respondents included family practitioners, internists, pediatricians, nurse practitioners, and physician assistants; all respondents were actively seeing patients in the United States and had been practicing for ≥3 years. We evaluated HCP features associated with (a) typically ordering diagnostic testing for tinea and (b) having seen, diagnosed, or consulted on cases of drug-resistant tinea using chi-square tests. We used t-tests to assess bivariate relationships and multivariable logistic regression with backward selection to estimate adjusted odds ratios (aOR) and 95% confidence intervals (CI).

## 3. Results

Among the 2591 HCPs invited to participate in the survey, 1753 (67.7%) completed it, and 1500 were asked the tinea questions. The HCPs were mainly internists (34.3%) or family practitioners (32.4%) in a group outpatient practice or clinic (64.9%) (Table 1).

Most HCPs (71.0%) said they diagnose tinea based on physical exam alone, whereas 20.1% typically use laboratory testing for tinea, most frequently with an in-office stain with microscopy (11.0%) (Table 2).

Overall, 19.5% of HCPs reported seeing, diagnosing, or consulting on cases of drug-resistant tinea, and 49.2% were aware of drug-resistant tinea but had not seen, diagnosed, or consulted on a case. Typically, performing diagnostic testing for tinea and clinical experience with drug-resistant tinea were highly correlated: 33.1% of the HCPs with clinical experience with drug-resistant tinea typically perform testing (vs. 16.8% of those without clinical experience) (*p* < 0.0001).

On multivariable analysis, the odds of typically performing diagnostic testing for tinea were significantly higher among HCPs who are Hispanic (aOR: 1.68, 95% CI: 1.05–2.69), are internists (aOR: 1.59, 95% CI: 1.12–2.28, reference group: family practitioners), see pediatric patients (aOR: 2.34, 95% CI: 1.64–3.35), see a higher number of patients per week (*p* = 0.002), or have teaching hospital privileges (aOR 1.60, 95% CI 1.21–2.10). The odds of having clinical experience with drug-resistant tinea were significantly higher among HCPs who were in an individual outpatient practice (aOR 1.94, 95% CI 1.18–3.17, reference group: inpatient practice/hospital) or a group outpatient practice (aOR 1.62, 95% CI 1.08–2.44), see pediatric patients (aOR 2.47, 95% CI: 1.74–3.52), see a higher number of patients per week (*p* = 0.0004), or have teaching hospital privileges (aOR 1.86, 95% CI: 1.42–2.45).

## 4. Discussion

In this survey of HCPs, one in five providers reported clinical experience with drug-resistant tinea, suggesting that this emerging issue may be more widespread in the United States than is represented by limited case reports [6,7]. However, a low proportion of HCPs reported typically performing diagnostic testing for tinea, indicating the potential for misdiagnosis and missed opportunities to detect antifungal drug-resistant cases. Together, the high reported familiarity with drug-resistant tinea and low testing could also indicate a possible misclassification of treatment failure as “drug-resistant tinea.”

The variability we observed in testing practices and experience with drug-resistant tinea may reflect differences in training and patient populations served. Factors influencing testing practices for tinea are also likely patient-related (i.e., demographic characteristics; site, severity, or clinical appearance of infection), which we did not evaluate. HCPs who see pediatric patients may be testing for tinea more often because tinea capitis is more common among children [8]. Possible reasons for the low testing rates include time constraints, lack of access to or unfamiliarity with in-house microscopy, or low insurance reimbursement rates. Social desirability bias may mean that testing for tinea is performed even less frequently than reported here. Conversely, HCPs may have overreported their familiarity with drug resistant tinea. A possible explanation for the relatively high reported rates of experience with drug-resistant tinea could be that providers were reporting tinea cases that failed treatment for reasons besides intrinsic antifungal resistance, such as incorrect diagnosis, improper treatment, or inadequate patient adherence to treatment [1]. In addition to potential misclassification of drug resistance, other limitations of this study include the lack of information about antifungal susceptibility testing and antifungal treatment practices.

Future work is needed to characterize the epidemiology of treatment-resistant tinea in the United States, using a One Health approach. The burden of treatment-resistant tinea is likely underestimated, particularly given providers’ reliance on visual inspection for tinea diagnosis and low rates of testing.

## Figures and Tables

**Table 1:** Healthcare-provider-related factors associated with performing diagnostic testing for tinea and reporting clinical experience with drug-resistant tinea, United States, 2022.

	Typically Perform Diagnostic Testing for Tinea			Have Seen, Diagnosed, or Consulted on Drug-Resistant Tinea Cases		
	Yes	No	*p*-Value	Yes	No	*p*-Value
	*n* = 301	*n* = 1199		*n* = 299	*n* = 1201	
**Demographic characteristics**						
Mean age in years (std dev)	46.8 (11.0)	46.1 (11.5)	0.318	46.9 (11.1)	46.2 (11.4)	0.462
Gender ^1^			0.046			0.900
Male	192 (64.2%)	685 (57.9%)		176 (59.5%)	701 (59.1%)	
Female	107 (35.8%)	499 (42.2%)		120 (40.5%)	486 (40.9%)	
Ethnicity			0.025			0.484
Non-Hispanic	273 (90.7%)	1130 (94.3%)		277 (92.6%)	1126 (93.8%)	
Hispanic	28 (9.3%)	69 (5.8%)		22 (7.4%)	75 (6.2%)	
Race			0.053			0.062
White	180 (59.8%)	813 (67.8%)		182 (60.9%)	811 (67.5%)	
Black or African American	14 (4.7%)	36 (3.0%)		13 (4.4%)	37 (3.1%)	
Asian	78 (25.9%)	262 (21.9%)		83 (27.8%)	257 (21.4%)	
Other	29 (9.6%)	88 (7.3%)		21 (7.0%)	96 (8.0%)	
Region			0.121			0.465
Northeast	65 (21.6%)	312 (26.0%)		70 (23.4%)	307 (25.6%)	
Midwest	82 (27.2%)	255 (21.3%)		73 (24.4%)	264 (22.0%)	
South	93 (30.9%)	379 (31.6%)		87 (29.1%)	385 (32.1%)	
West	61 (20.3%)	253 (21.1%)		69 (23.1%)	245 (20.4%)	
Metropolitan status			0.314			0.741
Urban	122 (40.5%)	431 (36.0%)		116 (38.8%)	437 (36.4%)	
Suburban	154 (51.2%)	652 (54.4%)		156 (52.2%)	650 (54.1%)	
Rural	25 (8.3%)	116 (9.7%)		27 (9.0%)	114 (9.5%)	
**Practice characteristics**						
Provider type			0.153			0.276
Family practitioner	97 (32.2%)	389 (32.4%)		107 (35.8%)	379 (31.6%)	
Internist	118 (39.2%)	396 (33.0%)		92 (30.8%)	422 (35.1%)	
Pediatrician	42 (14.0%)	208 (17.4%)		57 (19.1%)	193 (16.1%)	
Nurse practitioner	22 (7.3%)	82 (6.8%)		18 (6.0%)	86 (7.2%)	
Physician assistant	22 (7.3%)	124 (10.3%)		25 (8.4%)	121 (10.1%)	
Practice setting			0.107			0.004
Individual outpatient practice	57 (18.9%)	178 (14.9%)		54 (18.1%)	181 (15.1%)	
Group outpatient practice or clinic	195 (64.8%)	779 (65.0%)		207 (69.2%)	767 (63.9%)	
Inpatient practice/hospital	49 (16.3%)	242 (20.2%)		38 (12.7%)	253 (21.1%)	
See pediatric patients	236 (78.4%)	824 (68.7%)	0.001	254 (85.0%)	806 (67.1%)	<0.0001
Mean number of patients per week (std dev)	122.0 (81.4)	104.0 (69.5)	0.001	124.0 (75.7)	103.5 (71.0)	<0.0001
Teaching hospital privileges	174 (57.8%)	565 (47.2%)	0.001	172 (57.5%)	567 (47.2%)	0.001
Approximate household income of most patients			0.130			0.726
<USD 25,000	13 (4.3%)	85 (7.1%)		17 (5.7%)	81 (6.7%)	
USD 25,000–USD 49,999	76 (25.3%)	319 (26.6%)		71 (23.8%)	324 (27.0%)	
USD 50,000–USD 99,999	136 (45.2%)	493 (41.1%)		131 (43.8%)	498 (41.5%)	
USD 100,000–USD 249,999	60 (19.9%)	207 (17.3%)		56 (18.7%)	211 (17.6%)	
>USD 250,000	16 (5.3%)	95 (7.9%)		24 (8.0%)	87 (7.2%)	

^1^ seventeen providers responded “prefer to self-identify”.

**Table 2:** Diagnostic methods and experience with drug-resistant tinea, by healthcare provider type, United States, 2022.

What Methods Do You Typically Use to Diagnose Patients with Tinea (Ringworm) on Initial Presentation? ^1^	Family Practitioner *n* = 486	Internist *n* = 514	Pediatrician *n* = 250	Nurse Practitioner *n* = 104	Physician Assistant *n* = 146	Total *n* = 1500
In-office stain with microscopy	68 (14.0%)	69 (13.4%)	19 (7.6%)	13 (12.5%)	9 (6.2%)	178 (11.0%)
Fungal culture	40 (8.2%)	61 (11.9%)	32 (12.8%)	16 (15.3%)	11 (7.5%)	160 (10.7%)
Fungal PCR	24 (4.9%)	35 (6.8%)	7 (2.8%)	6 (5.8%)	11 (7.5%)	83 (5.5%)
Physical exam only/None of the above	380 (78.2%)	328 (63.8%)	199 (79.6%)	60 (57.7%)	98 (67.1%)	1065 (71.0%)
I do not see patients with tinea/ringworm	9 (1.9%)	68 (13.2%)	9 (3.6%)	22 (21.2%)	26 (17.8%)	134 (8.9%)
**Before this survey, were you familiar with reports of drug-resistant tinea (ringworm)?**						
Yes, I’ve seen, diagnosed, or consulted on cases of drug-resistant tinea (ringworm)	107 (22.0%)	92 (17.9%)	57 (22.8%)	18 (17.3%)	25 (17.1%)	299 (19.9%)
Yes, I’m aware of reports but have not seen, diagnosed, or consulted on a case	245 (50.4%)	244 (47.5%)	134 (53.6%)	44 (42.3%)	71 (48.6%)	738 (49.2%)
No	134 (27.6%)	178 (34.6%)	59 (23.6%)	42 (40.4%)	50 (34.3%)	463 (30.9%)

^1^ respondents could “select all that apply” for the first 3 answer choices.

## Data Availability

The CDC licensed the data for this study from Porter Novelli. The data are closed to the public but are available from Porter Novelli: https://www.porternovelli.com/.
